# Phylogenetics and systematics of the subfamilies Cheirurinae and Deiphoninae (Trilobita)

**DOI:** 10.1186/s13358-024-00338-1

**Published:** 2024-12-17

**Authors:** Francesc Pérez-Peris, Jonathan M. Adrain, Allison C. Daley

**Affiliations:** 1 Institute of Earth Sciences, University of Lausanne, Géopolis, 1015 Lausanne, Switzerland; 2 Present Address: Department of Earth and Environmental Sciences, University of Iowa, 115 Trowbridge Hall, Iowa City, IA 52242 USA

**Keywords:** Cladistics, Trilobites, Cheiruridae, Cheirurinae, Deiphoninae

## Abstract

Cheiruridae is one of the most diverse families of trilobites known from the Ordovician with 453 species assigned. Within Cheiruridae eight subfamilies (Acanthoparyphinae, Cheirurinae, “Cyrtometopinae”, Deiphoninae, Eccoptochilinae, Heliomerinae, Pilekiinae, and Sphaerexochinae) have historically been recognised. Insights about the evolution of the family and the relationships within and between subfamilies have been published. However larger scale phylogenetic hypotheses are needed in order to explore the monophyly, the basal structure, the deep nodes and the relationships of the subfamilies. Cheirurinae, Deiphoninae and “Cyrtometopinae” have historically been defined by various morphological features (e.g., anteroposterior constriction of the thoracic pleura, pleural furrow morphology, pygidial morphology) that differentiate them from the rest of Cheiruridae. However, the phylogenetic status of “Cyrtometopinae” is unclear owing to a lack of obvious synapomorphies. Here, we present phylogenetic analyses of Cheirurinae, Deiphoninae, and “Cyrtometopinae”. The results indicate that both Cheirurinae and Deiphoninae are monophyletic. “Cyrtometopines” are resolved as a paraphyletic grade at the base of Deiphoninae and Cyrtometopinae should be considered a junior subjective synonym of Deiphoninae. The new phylogenetic hypothesis reveals that paedomorphosis plays an important role in the evolution of Deiphoninae. Within Cheirurinae two major clades are identified, the ‘*Ceraurus*-like’ clade and the ‘*Ceraurinella*-like’ clade.

**Supplementary Information:**

The online version contains supplementary material available at 10.1186/s13358-024-00338-1.

## Introduction

The Family Cheiruridae Hawle & Corda, 1847 is a major clade of phacopide trilobites ranging from the latest Cambrian to the Middle Devonian. During the Ordovician the family was one of the most diverse trilobite groups with 453 described species and a worldwide distribution. The group has been extensively studied owing to its abundance in Ordovician and Silurian rocks and its broad morphological variability. The classification of Cheiruridae has a long history in the literature and is characterised by numerous changes in the systematics of the group (see Lane [1971] for a summary of the historical background). Within Cheiruridae eight subfamilies have been historically recognised: Acanthoparyphinae Whittington & Evitt, 1954, Cheirurinae Hawle & Corda, 1847, Cyrtometopinae Öpik, 1937, Deiphoninae Raymond, 1913, Eccoptochilinae Lane, 1971, Heliomerinae Evitt, 1951, Pilekiinae Sdzuy, 1955, and Sphaerexochinae Öpik, 1937. In addition, Prantl & Přibyl (1948) proposed the Subfamily Areiinae, which contains only the genus *Areia* Barrande, 1872. Areiinae shows strong morphological similarities with the subfamily Eccoptochilinae and its validity is uncertain, although some authors have recognised it (e.g., Lane, 1971).

Relationships within Cheiruridae are unclear, probably owing to the morphological diversity across the family and a lack of phylogenetic analyses that have included representatives of the whole family. Acanthoparyphinae (Adrain, 1998), Deiphoninae (Congreve & Lieberman, 2010), Eccoptochilinae (Gapp et al., 2012) and Sphaerexochinae (Congreve & Lieberman, 2011) have been subjected to phylogenetic analyses, but each of these studies focused on relationships within, not between, subfamilies. Consequently, the branching order of the major subgroups is unknown, as well as the relationships between them. Indeed, even the monophyletic status of some of the subfamilies has not been well established (e.g., Pilekiinae; Adrain & Karim, 2019). The subfamilies Heliomerinae, Sphaerexochinae and Pilekiinae are especially problematic regarding their relationships with the rest of the Cheiruridae. Each of these subfamilies is characterised by distinctive morphologies, different from the rest of cheirurids, making it difficult to determine their affinities. As a consequence, they have experienced numerous changes in their taxonomy. Recent work (Adrain & Karim, 2019; Adrain & Pérez-Peris, 2021) clarified the apomorphies of some cheirurid subfamilies (e.g., Acanthoparyphinae, Sphaerexochinae, Pilekiinae) and future work should address the internal relationships of the family.

**Fig. 1:**
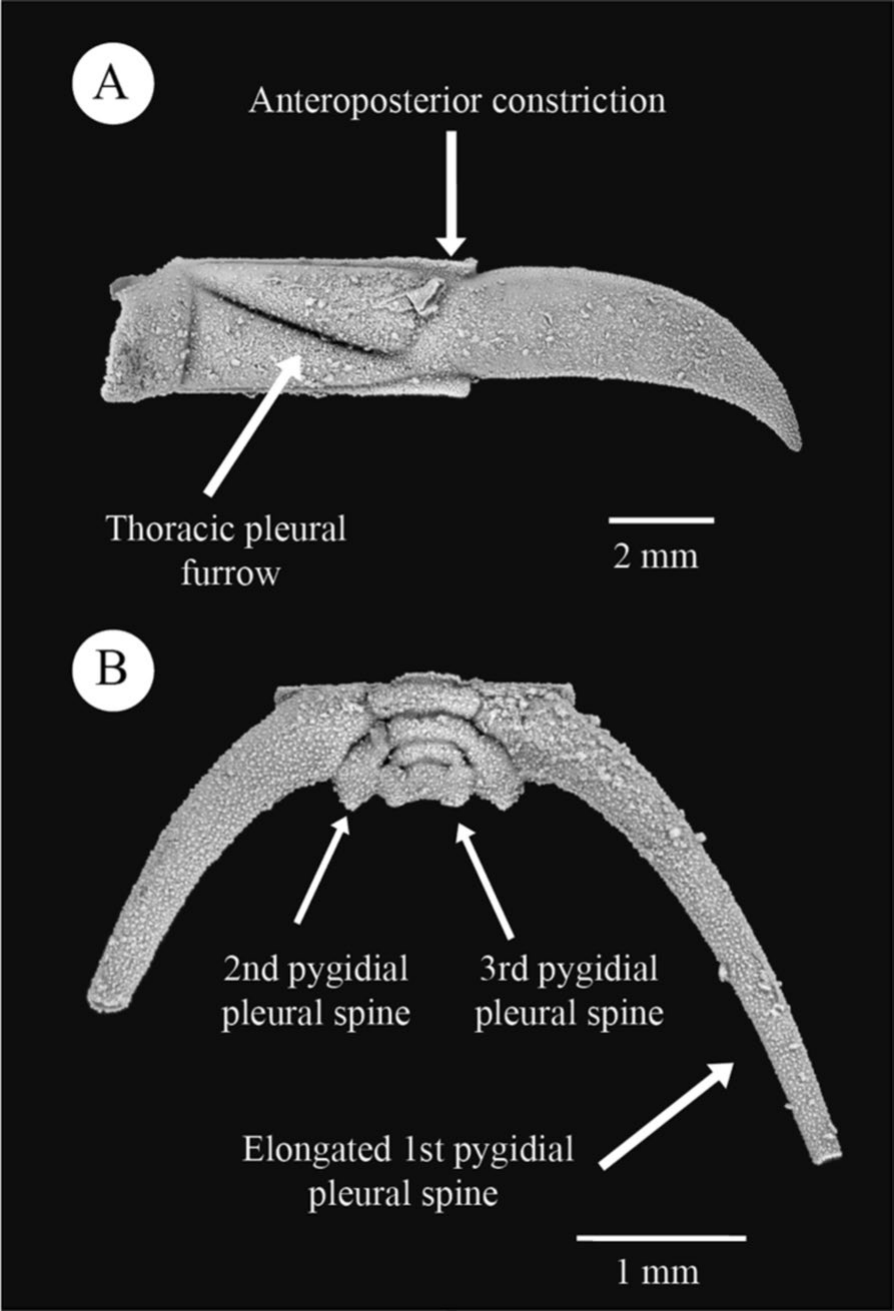
Thoracic pleural and pygidial morphology of *Laneites polydorus,* from the Table Cove Formation, western Newfoundland, Canada*.* **A** Thoracic pleura in dorsal view. The anteroposterior constriction is indicated. **B** Pygidium in dorsal view. The three pairs of pygidial pleural spines with the first pair elongated are indicates. Specimens GSC 135177 (**A**) and GSC 135184 (**B**) (GSC = Geological Survey of Canada). All specimens figured by Adrain and Pérez-Peris (2021).

Among all cheirurid subfamilies, Cheirurinae, “Cyrtometopinae” and Deiphoninae share several unique morphological characters, suggesting close relationship. The two most noticeable are the abaxial anteroposterior constriction of the thoracic pleura and the well-developed fulcral process and fulcral socket (Fig. 1A). Another significant similarity between the subfamilies is the pygidial configuration. This is particularly important in the oldest members of each subfamily (e.g., *Krattaspis* Öpik, 1937 and *Cyrtometopus* Angelin, 1854 in “Cyrtometopinae”, *Mainbrookia* Adrain & Pérez-Peris, 2021 and *Hemisphaereocoryphe* Reed, 1896 in Deiphoninae and *Sycophantia* Fortey, 1980 in Cheirurinae). In these taxa the pygidium is composed of three axial rings and a terminal piece (which can be expressed or not), an elongate first pair of pygidial pleural spines and reduced second and third pairs (Fig. 1B). These morphological characters could be considered putative synapomorphies of a larger clade composed of the three subfamilies.

The definition and content of the Subfamily “Cyrtometopinae” has been problematic ever since Öpik (1937) proposed the group. Öpik (1937) defined the subfamily based on the possession of a transverse thoracic pleural furrow, or row of pits, parallel to the anterior and posterior margins of the pleura. Öpik (1937) included thirteen genera: *Cyrtometopus*, *Eccoptochile* Hawle & Corda, 1847, *Pseudosphaerexochus* Schmidt, 1881, *Actinopeltis* Hawle & Corda, 1847, *Nieszkowskia* Schmidt, 1881, *Hemisphaerocoryphe*, *Pilekia* Barton, 1915, *Youngia* Lindström, 1885, *Anacheirurus* Reed, 1896, *Kawina* Barton, 1915, *Parapilekia* Kobayashi, 1934, *Seisonia* Kobayashi, 1934, *Reraspis* Öpik, 1937 and *Protopliomerops* Kobayashi, 1934. Most of these genera are now classified in other subfamilies. The transverse thoracic pleural furrow is a feature widespread across the whole of Cheiruridae, present in acanthoparyphines (Adrain & Pérez-Peris, 2021), deiphonines (Lane, 1971), pilekiines (Pérez-Peris et al., 2021) and eccoptochilines (Gapp et al., 2012), making it a broadly distributed symplesiomorphy. As a result, “Cyrtometopinae” was composed of a collection of trilobites with a great range of morphological variability. Lane (1971) questioned the validity of the subfamily and split “cyrtometopines” into two groups. Taxa with the anteroposterior pleural constriction (*Cyrtometopus*, *Reraspis*, *Eccoptochiloides* Prantl & Přibyl, 1948, *Actinopeltis* and *Zazvorkaspis* Přibyl & Vaněk, 1964) were assigned to Cheirurinae, and the rest were placed in a new subfamily, Eccoptochilinae. Subsequently, some authors (e.g., Pärnaste, 2003; Přibyl et al., 1985) revived the concept of “Cyrtometopinae” based on taxa with the anteroposterior pleural constriction. Pärnaste (2003) included *Sphaerocoryphe* and *Hemisphaerocoryphe* within “Cyrtometopinae”. However, most of the suggested apomorphies of this version of “Cyrtometopinae” are features shared with Deiphoninae.

The aim of this work is to produce the first phylogenetic analyses that include the cheirurid subfamilies Cheirurinae, Deiphoninae and “Cyrtometopinae”, in order to unravel their inter- and intra-relationships, define their deep nodes, test their monophyly and understand the early stages of their evolution.

## Material and methods

*Taxa selected*—A total of 39 Ordovician species from 27 genera were included in the analyses (information about all taxa analysed is included in Appendix I), encompassing most of the Ordovician cheirurine, deiphonine and “cyrtometopine” generic diversity. Six genera (*Hinggania* Zhao in Zhao et al., 1997, *Hapsiceraurus* Whittington, 1954*, Junggarella* Xiang and Zhang in Zhang T., 1981, *Lonchocheirurus* Zhou Z.-Q., Zhou Z. -Y., and Xiang, 2016*, Osekaspis* Prant and Přibyl, 1948 and *Zazvorkaspis*) were excluded from the analyses because limited morphological information is available for them. For each genus, species with the most complete morphological record were selected. Where possible, more than one species were selected for genera with high diversity (e.g., *Ceraurinella*, *Ceraurinus* Barton, 1913*, Ceraurus* Green, 1832, *Cyrtometopus, Gabriceraurus* Přibyl and Vaněk in Přibyl et al., 1985, *Sphaerocoryphe)*. Finally, in cases within a genus where there is an older assigned species clearly morphologically distinct from its congeners, that older species was included (e.g., *Acticopeltis completa* [Barrande, 1864], *Ceraurinella magnilobata* Tripp, 1967*, Gabriceraurus proicens* [Tripp, 1967]), even if incompletely known.

*Outgroup selection*—Pilekiines have long been considered basal to the rest of the cheirurid subfamilies (e.g., Whittington, 1966, text fig.-10; Lane, 1971, text-figs. 10, 12, 13). Adrain and Karim (2019) reviewed the cheiruroidean affinities of pilekiines and pointed out: “Pilekiinae has generally been conceived of as consisting of early, plesiomorphic species of Cheiruridae which lack the synapomorphies of other subfamilies”. Here we follow the hypothesis of pilekiines as sister taxa of the rest of cheirurid subfamilies. Within Pilekiinae, *Parapilekia oleasnensis* (Růžička, 1935), from the upper Tremadocian of Milina Formation, Czech Republic, was chosen as an outgroup. *Parapilekia oleasnensis* is one of the best known pilekiine species, with full articulated specimens and information on the morphology of the hypostome.

**Fig. 2:**
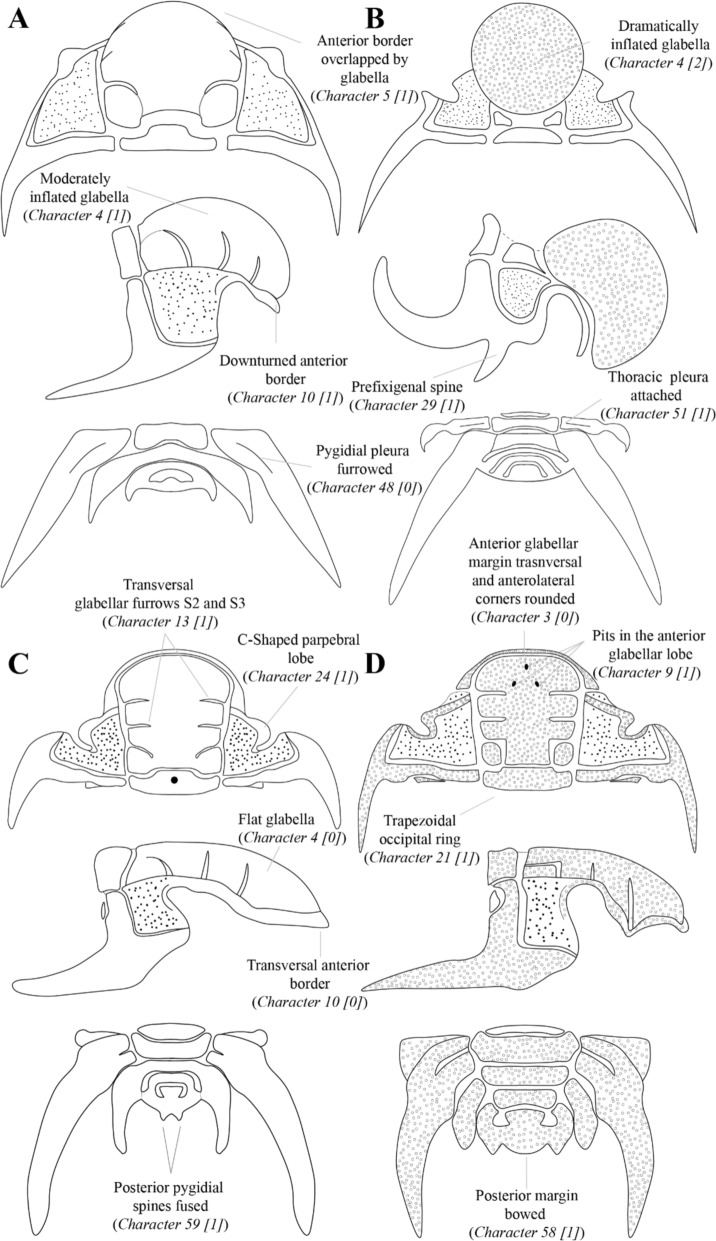
Schematic drawing of species belonging to Deiphoninae: **A** *Cyrtometopus affinis* **B** *Sphaerocoryphe ludvigseni* and Cheirurinae: **C** *Ceraurinella nahanniensis* **D** *Gabriceraurus gabrielsi*. For each species, drawings represent (from top to bottom) the cranidium in dorsal view, the cranidium in lateral view, and the pygidium in dorsal view. Main morphological characters are indicated

*Characters*—A total of 64 characters were selected, of which 33 describe the cranidium, one the rostral plate, six the hypostome, seven the thorax, 16 the pygidium and one the thoracopygidium trunk (see Fig. 2 for schematic drawings of some of the characters). Characters were coded using reductive coding sensu Strong and Lipscomb (1999) and all characters were treated as unordered. Characters 22 (Position of the eye), 23 (Relative length [exsag.] of the palpebral lobe), 40 (Relative length [sag.] of the hypostome middle body) and 42 (Relative width [tr.] of the proximal part of the thoracic pleura) are based on continuous data, but were coded as discrete character states. The data were divided by the length (exsg.) of the fixigena for characters 22 and 23, the width (tr.) of the middle hypostome body for character 38 and the width (tr.) of the thoracic axial rings for character 43. Subsequently, based on the distribution of data, different character states were created that represent ranges of the obtained ratios (raw data from the measurements and histograms showing the data distribution are included in Appendix II).

Character 47 (maximum number of thoracic segments) and character 63 (maximum number of axial rings in the pygidium in holaspides) are problematic because of the differences in the number of segments allocated to the thoracopygidium, thorax and pygidia across the taxa analysed. Most of the species analysed display a configuration of 14 segments in the thoracopygidium, with 11 segments in the thorax and 3 in the pygidium. However, within the deiphonines some genera display 13 segments in the thoracopygidium, with 10 segments in the thorax and 3 in the pygidium, or 9 and 4 respectively. Here it is considered that the pygidium and thorax are homologous structures across all the taxa, with the exception of the genus *Sphaerocoryphe*. The clear thoracic morphology of the first pygidial segment, together with the developmental series of the genus (segments are allocated in the protopygidium and posteriorly released into the thorax; Tripp et al., 1997), suggest that the first segment in the pygidium of *Sphaerocoryphe* is not homologous to the first pygidial segment of the rest of genera. Instead the second segment, which bears an elongated pleural spine, was counted as homologous to the first pygidial segment of all the rest of the species.

### Character list

**Cranidium**:

1. Orientation of axial furrow from the occipital furrow to S3

0: anteriorly convergent; 1: parallel; 2: anteriorly divergent; 3: laterally bowed.

2) Size of the anterior lobe of the glabella

0: similar in size to the rest of the glabellar lobes; 1: greatly enlarged, clearly distinct from the rest of glabellar lobes.

3) Shape of the anterior glabellar lobe

0: suboval, with anterior margin transverse or almost transverse in medial area, anterolateral corners rounded (Fig. 2D); 1: semicircular with anterior margin greatly bowed anteriorly; 2: subrectangular, with anterior margin transverse (perpendicular to the sagittal axis) and anterolateral corners relatively pointed; 3: subtrapezoidal, with medial part of the anterior margin transverse (perpendicular to the sagittal axis), anterolateral corners slightly rounded and lateral side of the anterior margin sloping posterolaterally.

4) Degree of inflation of the glabella

0: flat to slightly inflated (Fig. 2C); 1: moderately inflated (Fig. 2A); 2: dramatically inflated (Fig. 2B).

5) Relative position of anterior margin of glabella and anterior border

0: anterior margin of glabella posterior to rear of anterior border in dorsal view (Fig 2C, D); 1: anterior margin of glabella obscures part or all anterior border in dorsal view (Fig 2A, B).

6) Glabellar sculpture

0: smooth or tiny granules; 1: medium granules; 2: coarse tubercles.

7) Density of glabellar tuberculation

Inapplicable for taxa coded as 0 and 1 in character 6.

0: sparse arranged; 1: densely arranged.

8) Paired glabellar tubercles in mature holaspides

Inapplicable for taxa coded as 0 and 1 in character 6.

0: absence of prominent paired tubercles; 1: presence of prominent paired tubercles.

9) Pits on the anterior glabellar lobe

0: absent; 1: pits arranged in a triangle, with two posterior pits in exsagittal position and oneanterior pit in a sagittal position (Fig. 2D).

10) Slope of the anterior border

0: horizontal (Fig. 2C); 1: Downturned anteriorly (Fig. 2A).

11) Definition of the anterior border furrow laterally, separating the anterior border of the cranidium from the fixigena

0: highly effaced; 1: well defined.

12) Definition of glabellar furrows S2 and S3

0: effaced or highly effaced; 1: well defined.

13) Orientation of glabellar furrows S2 and S3

Inapplicable for taxa coded as 0 in character 12.

0: running posteromedially; 1: transverse (Fig. 2C).

14) Width (tr.) of S2 and S3

Inapplicable for taxa coded as 0 in character 12.

0: narrow (tr.), reduced to the glabellar margin; 1: wide (tr.) at least one quarter of the glabellar width.

15) Course of S1

0: separate right and left furrows, not connected medially; 1: transglabellar, connected medially across the glabella.

16) Depth of the transglabellar S1

Inapplicable for taxa coded as 0 in character 15.

0: shallow, expressed as a faint depression; 1: deeply incised, well defined.

17) Orientation of S1 distally, adjacent to the axial furrow

0: posteriorly directed; 1: transversely directed.

18) Orientation of S1 proximally

Inapplicable for taxa coded as 1 in character 15.

0: S1 bending posteriorly running posteromedially; 1: S1 bending posteriorly, running posteriorly parallel to the sagittal axis.

19) Width (tr.) of S1 compared to S2 and S3

0: S3 and S2 same width (tr.) as S1; 1: S3 and S2 narrower (tr.) than S1; 2: S3 and S2 wider (tr.) than S1.

20) Shape of the posterior margin of the glabella

0: bowed anteriorly at medial part; 1: transverse;

21) Shape of the occipital ring

0: rectangular; 1: sub-trapezoidal, with anterior margin medially transversal (Fig. 2C); 2: bowed anteriorly, describing a forwardly directed arch.

22) Position of the eye

0: anterior (ratio of distance from the posterior tip of the palpebral lobe to the posterior cephalic margin/maximum fixigenal length (exsag.) ≥ 0.45); 1: medial (ratio < 0.45 and > 0.25); 2: posterior (ratio ≤ 0.25).

23) Relative length (exsag.) of the palpebral lobe

0: short (ratio of the palpebral lobe length (exsag.) / maximum fixigenal length (exsag.) < 0.40; 1: long (≥ 0.40).

24) Shape of the palpebral lobe

0: suboval, tear drop shaped; 1: deflected adaxially posteriorly, laterally convex, C-shaped (Fig. 2C).

25) Definition of the eye ridge in holaspid

0: absent or not well defined by a furrow; 1: well defined by a furrow.

26) Presence of sutural ridge along the anterior section of the facial suture, continuous with the anterior border of the cranidium.

0: absent; 1: present.

27) Course of the eye ridge

0: Eye ridge located at the anteriormost part of the fixigenal field with most of the anterior margin running along the anterior branch of the facial suture; 2) Eye ridge crosses the fixigenal field, not running along the anterior branch of the facial suture, isolating an anterior area of the fixigenal field.

28) Size of the genal spine

0: genal spine reduced, not exceeding the maximum length (sag.) of the cranidium; 1: genal spine elongate, with a stout base at the genal angle, exceeding the maximum length (sag.) of the cranidium.

29) Presence of prefixigenal spines in mature holaspides

0: absent; 1: present (Fig. 2B).

30) Number of prefixigenal spines

Inapplicable for taxa coded as 0 in character 27.

0: one; 1: two.

31) Tubercles on librigenal field

0: absent; 1: present.

32) Presence of two tubercles on the posterior part of the fixigena, exsagittally aligned with the palpebral lobe

0: absent; 1: present.

33) Direction of the posterior branch of the facial suture from the posterior tip of the palpebral lobe (ε) to the lateral border furrow (ω)

0: posterolateral; 1: transverse; 2: anterolateral.

**Rostral plate**:

34) Rostral plate bearing a pair of spines anteriorly projected

0: absent; 1: present.

**Hypostome**:

35) Anterior lobe of middle body underhanging anterior border in ventral view

0: absent; 1: present.

36) Shape of anterior border

0: slightly bowed anteriorly, anterior wings transverse to slightly turned posteriorly; 1: greatly bowed anteriorly, anterior wings turned posteriorly; 2: transverse medially, anterior wings turned forward abaxially.

37) Definition of maculae

0: well defined; 1: not well defined.

38) Shape of posterior border

0: posteriorly bowed; 1: transverse; 2: slightly anteriorly bowed.

39) Pair of posteriorly directed spines in the posterior border of the hypostome

0: absent; 1: present.

40) Relative length (sag.) of middle body

0: Elongated (maximum length (sag.)/maximum width (tr.) ≥ 1.2): 1: nonelongated (maximum length (sag.)/maximum width (tr.) < 1.2).

**Thorax**:

41) Anteroposterior exsagittal constriction of the thoracic pleura
  0: absent; 1: present.
42) Relative width (tr.) of proximal part of thoracic pleura

0: long (width (tr.) of the proximal part of the thoracic pleura / width (tr.) of the axial ring x ≥ 0.75); 1: medium (0.4 < x < 0.75); 2: short (x ≤ 0.4).

43) Orientation of pleural furrow

0: running slightly obliquely from the anterior corner of the pleura, straight all along the furrow; 1: transverse; 2: oblique adaxially and transverse abaxially; 3: oblique adaxially and slightly curved abaxially.

44) Type of pleural furrow

The pleural furrow of *Reraspis plautini* has been considered as a continuous furrow. Following the interpretation of Pärnaste (2004), the interruptions in the furrow are abnormalities or artefacts of preservation.

0: continuous furrow; 1: pitted furrow.

45) Pleural furrow turns forwards abaxially (exsag.) at the fulcrum forming an anteroposterior furrow

Inapplicable for taxa coded as 0 in character 44.

0: absent; 1: shallow, as a small depression; 2: well defined.

46) Paired tubercles on axial ring in mature holaspids

0: absent; 1: present.

47) Maximum number of thoracic segments in holaspid

In *Sphaerocoryphe* the first pygidial segment in the thorax is not counted, despite its thoracic morphological similarity.

0: fourteen; 1: eleven; 2: ten; 3: nine.

**Pygidium**:

48) Present of pleural furrow on first pygidial segment

0: present; 1: absent.

49) Dorsal inflation of pygidial axial rings:

0: axial rings inflated dorsally, first ring most inflated, in inflation gradually decreasing posteriorly; 1: first ring inflated, posterior rings flat and extremely reduced, creating flat depression between the first axial ring and the pygidial rim.

50) Second pygidial axial ring separated by axial furrow from the pleural field

0: present 1: absent.

51) First pygidial segment is an undetached thoracic segment

0: absent; 1: present.

52) Size of the articulating flanges of the anterior margin of the pygidium

0: small to medium size, never wider (tr.) than the maximum pygidial width (tr.); 1: laterally prominent, being wider (tr.) than the rest of the pygidium.

53) Presence of macropleural pygidial spines

0: absent; 1: present.

54) Presence of carina on first pair of pleural spines

0: absent; 1: present.

55) Degree of fusion of pygidial pleural field

0: not fused, with the pleural segments and pleural spines recognizable; 1: fused with the interpleural furrows effaced forming a pygidial margin.

56) Size of second pygidial pleural spine

In the genus *Sphaerocoryphe* the second pygidial pleural spine is counted as the spine posterior to the elongate pygidial spines.

0: long, well differentiated from the pygidial margin; 1: short, as a small triangular notch in the pygidial margin; 2: absent.

57) Size of last pair of pygidial spines

0: long, well developed; 1: Short, triangular notches; 2: absent.

58) Shape of posterior margin of pygidium between the last pair of pygidial spines

0: transverse; 1: posteriorly bowed; 2: extended into a spine (Fig. 2D).

59) Fusion of posteriormost pleural spines

0: absent; 1: present (Fig. 2C).

60) Degree of fusion of posterior pair of pygidial pleural spines

Inapplicable for taxa coded as 0 in character 59.

0: partially fused, with tip of the spine visible; 1: completely fused.

61) Degree of differentiation of the terminal piece

0: well differentiated; 1: almost not differentiated or absent;

62) Pair of ventral projections on the pygidial rim

0: absent; 1: present.

63) Number of pygidial axial rings in holaspides

0: five; 1: four; 2: three.

**Toracopygidium**:

64) Number of segments in the trunk in holaspides

0: nineteen; 1: fifteen; 2: fourteen; 3: thirteen.

*Analyses*—Parsimony analyses were performed in TNT version 1.5 (Goloboff & Catalano, 2016) using the heuristic “Traditional Search” (1000 replicates × 1000 iterations). TBR was used for the branch-swapping algorithm. Initially all characters were treated as unordered and equally weighted. In subsequent analyses characters were reweighted using implied weights. A concavity of K = 12 was selected for the analysis, following Goloboff et al., (2018), who showed it performs better than lower values. Nodal support was calculated, using GC bootstrap (1000 reps.; Goloboff et al., 2003), jacknife (1000 reps.) and Bremer support values. Under implied weights, the nodal support was calculated using symmetric resampling (G/C values). The character matrix coded is available in Appendix III.

The tree topology from the strict consensus was plotted against the geologic time scale using R package strap (Bell & Lloyd, 2015). The stratigraphic ranges of the species were used to produce a stratigraphic-calibrated tree. Such stratigraphic ranges are approximate due to the lack of temporal constrain in most of the species stratigraphic distribution and the uncertainties in global correlations. Consequently, most of the species display a broader stratigraphic distribution that their actual range.

## Results

**Fig. 3:**
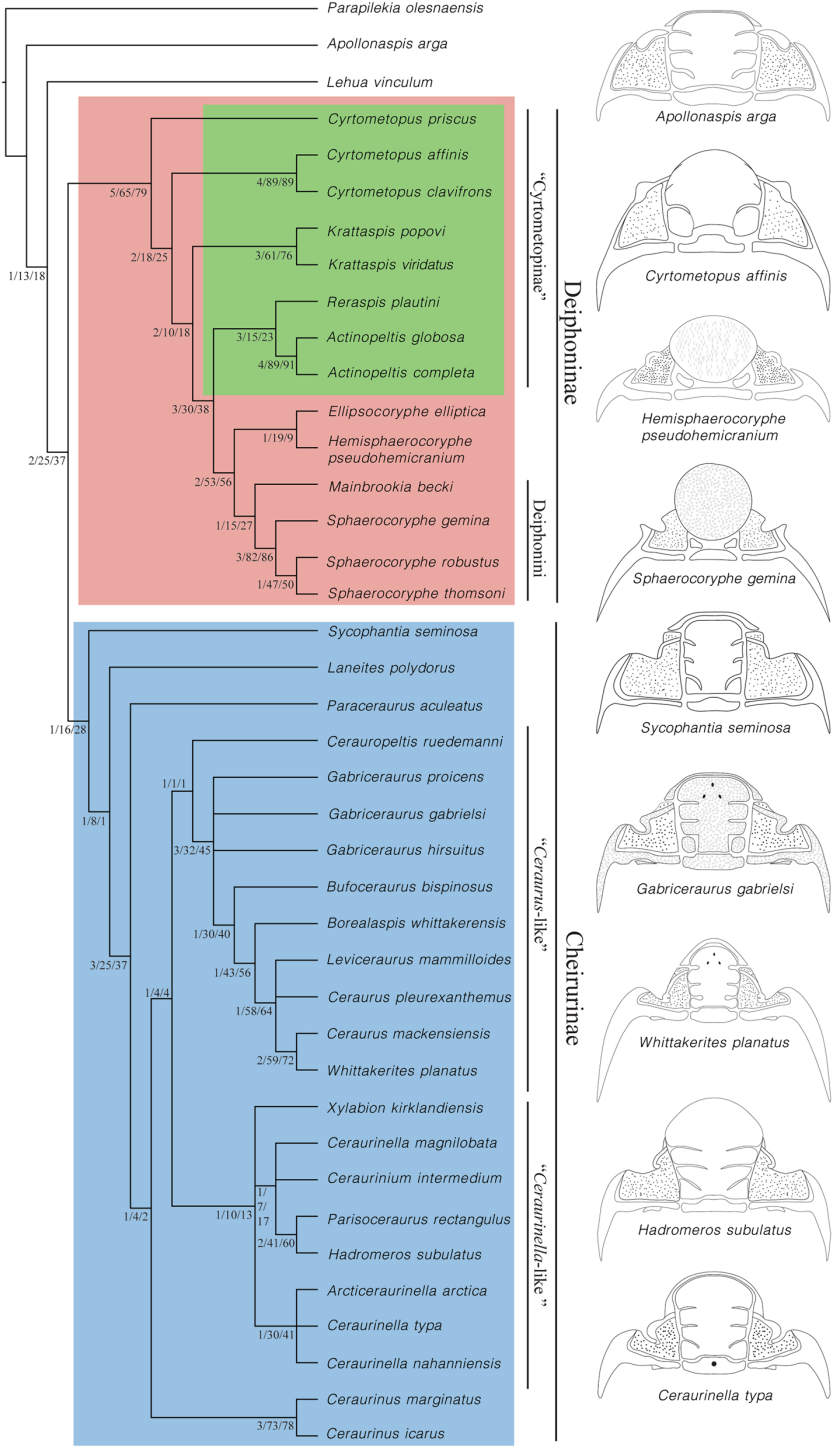
Strict consensus tree of the 27 most parsimonious trees obtained from the parsimony analysis under equal weights. Nodes numbers indicate Bremer, bootstrap and jacknife supports. Highlighted in green are the nodes of the tree assigned to “Cyrtometopinae”, in orange the nodes assigned to Deiphoninae and in blue the nodes assigned to Cheirurinae.

**Fig. 4:**
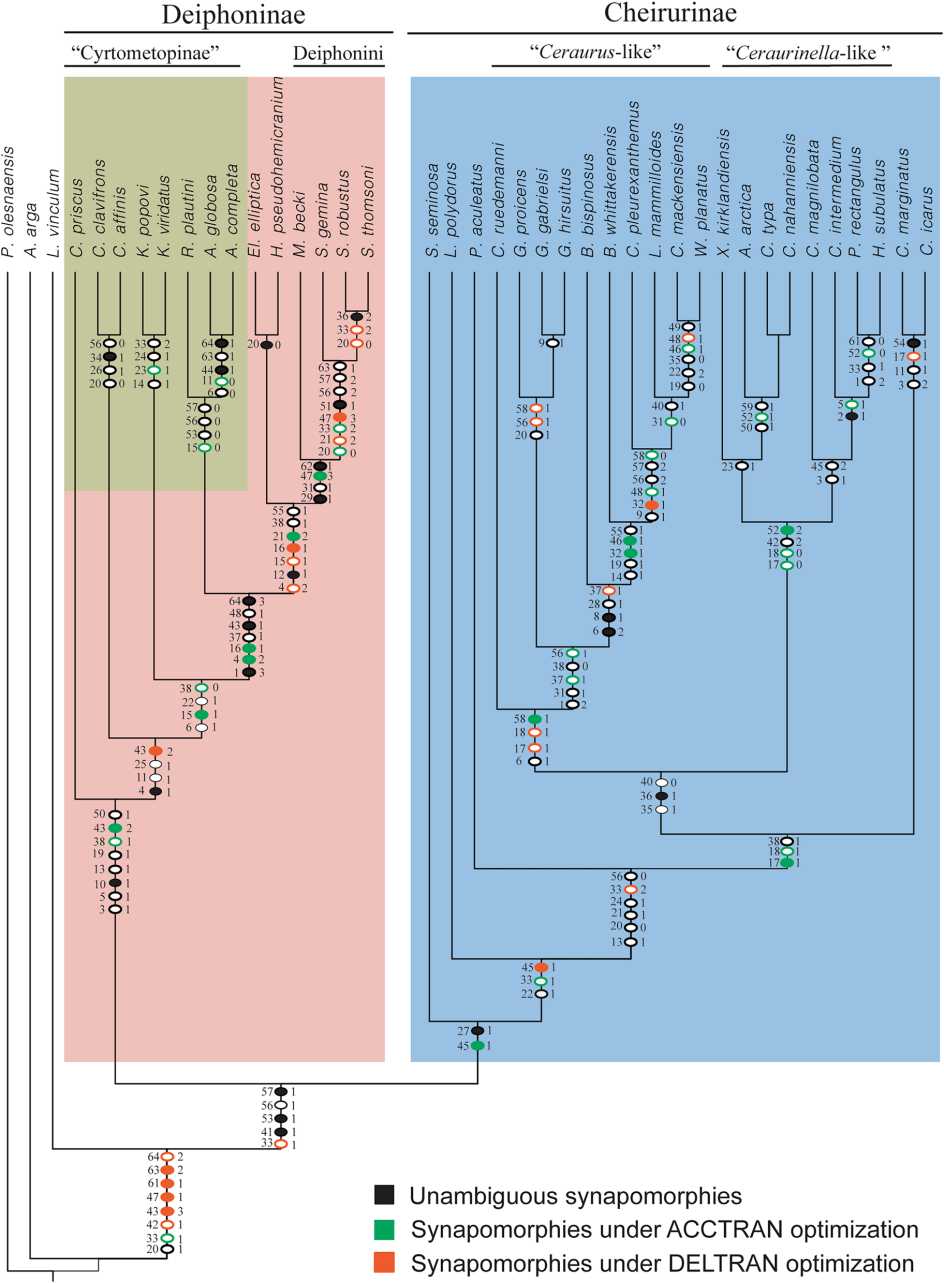
Character optimization. Unambiguous synapomorphies (black), synapomorphies under ACCTRAN optimization (green) and synapomorphies under DELTRAN optimization (orange), where the number on the left of each dot is the character number and the number on the right the character state. Open circles indicate characters containing homoplasy. Highlighted in green are the nodes of the tree assigned to “cyrtometopinae”, in orange the nodes assigned to Deiphoninae and in blue the nodes assigned to Cheirurinae.

**Fig. 5:**
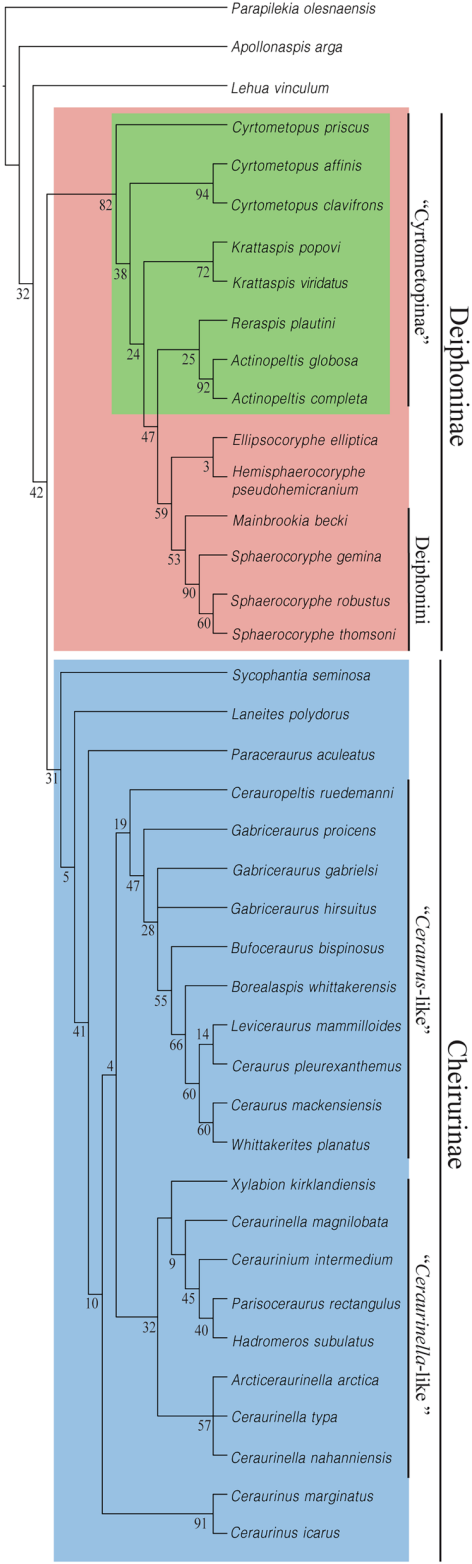
Strict consensus tree of the two most parsimonious trees recovered under parsimony analysis with implied weights (K = 12). Node numbers obtained from symmetrical resampling. Highlighted in green are the nodes of the tree assigned to “Cyrtometopinae”, in orange the nodes assigned to Deiphoninae and in blue the nodes assigned to Cheirurinae.

Parsimony analysis under equal weights recovered 27 most parsimonious trees (MPTs) each of 237 steps, CI (Consitency index) of 0.36 and RI (Retention Index) of 0.69 (Fig. 3). Unambiguous synapomorphies (Fig. 4) and synapomorphies optimised using the accelerated transformation (ACCTRAN; Fig. 4) and delayed transformation (DELTRAN; Fig. 4) assumptions for all nodes are shown on one of the most parsimonious trees. The topology was selected following the maximal stratigraphic congruence criteria. Analysis with implied weights (K = 12) recovered 2 MPTs each with 50.96 fit, CI 0.37 and RI = 0.70 (Fig. 5).

Both consensus trees, under equal weights and under implied weights, are highly resolved (Figs. 3, 5), and highly congruent with one another. Two clades corresponding to the subfamilies Cheirurinae and Deiphoninae are recovered in all analyses. The major clade Cheirurinae + Deiphoninae is defined by the anteroposterior constriction of the thoracic pleura (Character 41 [1]) and the morphology of the pygidium, which is characterised by a first pair of elongated pygidial pleural spine (Character 53 [1]) followed by a reduced second and third pair (Characters 56 [1] and 57 [1]) (Fig. 4). DELTRAN optimization suggests as a putative synapomorphy of the group a transversally directed posterior branch of the facial suture (Character 33 [1]; Fig. 4). The species *Lehua vinculum* (Barrande, 1872) and *Apollonaspis arga* (Whittington, 1963) are resolved outside the Cheirurinae + Deiphoninae clade, being the former sister taxon to the major clade.

Deiphoninae shows high support values in both analyses (Figs. 3, 5). The synapomorphies that define Deiphoninae are: semi-circular, greatly anteriorly bowed anterior margin of the glabella (Character 3 [1]), anterior border of the cranidium dorsally overlapped by the glabella (Character 5 [1]), anterior cranidial border sloping downwards from horizontal (Character 10 [1]), narrow (tr.) transverse S2 and S3 (Characters 13 [1] and 19 [1]) and second axial ring of the pygidium not differentiated from the pygidial pleura (Character 50 [1]) (Fig. 4). No unambiguous synapomorphies are defined based on trunk or hypostome morphology, probably due to the absence of data for both in *Cyrtometopus priscus* Tjernvik, 1956, the earliest-diverging member within Deiphoninae. However, ACCTRAN optimization suggests as other possible synapomorphies of the clade the transverse posterior border of the hypostome (Character 38 [1]) and the proximally oblique and distally transverse pleural furrow (Character 43 [2]) (Fig. 4).

Within Deiphoninae the topology is conserved across unweighted and weighted trees. “Cyrtometopines” are resolved as a paraphyletic grade at the base of Deiphoninae, with *Cytometopus priscus* as the earliest-diverging taxon within the clade (Figs. 3, 5). *Reraspis* and *Actinopeltis* form a clade characterised by an homonomous trunk with a pygidium bearing equally developed pygidial spines (Characters 53 [0], 56 [0], 57 [0]). This clade is the sister taxon of a major group historically considered deiphonines (*Ellipsocoryphe* Lu, 1975, *Hemisphaerocoryphe*, *Mainbrookia* and *Sphaerocoryphe*), which shows relatively high support values (Figs. 3, 5) The synapomorphies that define the historically recognised deiphonines are the effacement of S2 and S3 (Character 12 [1]), a transverse posterior margin of the hypostome (Character 38 [1]) and fusion of the pygidial pleural bands through the effacement of the interpleural furrows (Character 55 [1]) (Fig. 4). Other putative synapomorphy of the group resolved under ACCTRAN optimization is an anteriorly bowed anterior margin of the occipital ring (Character 21 [2]) (Fig. 4). Under DELTRAN optimization, a dramatically inflated glabella (Character 4 [2]), a transglabellar and deeply incised S1 (Characters 15 [1], 16 [1]) are recovered as putative synapomorphies (Fig. 4). Two clades within the historical deiphonines are recovered, one formed by *Ellipsocoryphe* and *Hemisphaerocoryphe*, and a second that includes the genera *Mainbrookia* and *Sphaerocoryphe*.

Cheirurinae is defined by having an eye ridge that runs across the anterior part of the fixigena and not along the anterior branch of the facial suture (Character 27 [1]) (Fig. 4). In addition, ACCTRAN optimization suggests as a putative synapomorphy for the subfamily the pleural furrow distally curving forward (Character 45 [1]) (Fig. 4). However, the earliest-diverging taxon within the subfamily is *Sycophantia seminosa* Fortey, 1980, for which thoracic information is missing, and the hypostome attributed to it (Fortey, 1980, pl. 16, figs. 3, 6) has been suggested to be a pliomerid hypostome (Adrain & Pérez-Peris, 2021, p. 7). Contrary to Deiphoninae, Cheirurinae node shows low support values (Figs. 3, 5). Within the subfamily two major clades are recovered (Figs. 3, 5), one containing ‘*Ceraurus*-like’ forms and another containing ‘*Ceraurinella*-like’ forms. Both clades have low support values at their early node (Figs. 3, 5). All the synapomorphies of the *Ceraurus*-like + *Ceraurinella*-like clade are based on hypostomal characters: the middle body overlaps the anterior border of the hypostome (Character 35 [1]), the anterior margin is greatly bowed forward (Character 36 [1]) and the middle body is relatively long (Character 40 [0]). *Sycophantia seminosa*, *Laneites polydorus* (Billings, 1865) and *Paraceraurus aculeatus* (Eichwald, 1860) are resolved at the base of the clade and the genus *Ceraurinus* is resolved as sister taxa to the ‘*Ceraurus*-like’ + ‘*Ceraurinella*-like’ clade.

The ‘*Ceraurus*-like’ clade is unambiguously supported by granular glabellar sculpture (Character 6 [1]) (Fig. 4). In addition, under DELTRAN optimization (Fig. 4) two other synapomorphies are suggested: an S1 that is transverse distally (Character 17 [1]) and parallel to the sagittal axis medially (Character 18 [1]). ACCTRAN optimization (Fig. 4) suggests a posteriorly bowed posterior margin of the pygidium (Character 58 [1]) as another potential synapomorphy of the clade. The topology of the clade is almost identical under equal weights and under implied weights (Figs. 3, 5). *Cerauropeltis ruedemani* (Raymond, 1916) is the earliest-diverging taxon within the ‘*Ceraurus*-like’ clade. All the species from the genus *Gabriceraurus* are resolved in a polytomy together with a major clade containing all the rest of members of the ‘*Ceraurus*-like’ group. Under implied weights, *Gabriceraurus proicens* is resolved as sister taxon to this clade. Under implied weights *Leviceraurus mammiloides* Hessin, 1998 and *Ceraurus pleurenxanthemus* Green, 1832 form a clade which is sister to *Whittakerites planatus* Ludvigsen, 1976 and *Ceraurus mackensiensis* Ludvigsen, 1979*.*

The ‘*Ceraurinella*-like’ clade is defined by a narrow (tr.) proximal part of the thoracic pleura (Character 42 [2]) (Fig. 4). ACCTRAN optimization (Fig. 4) suggests the shape of S1 and the size of the pygidial flanges as putative synapomorphies (Characters 17 [0], 18 [0] and 52 [2]). Two major clades within the ‘*Ceraurinella*-like’ group are resolved, one containing the genera *Ceraurinella* and *Arcticeraurinell*a Přibyl and Vaněk in Přibyl et al., 1985 and the other the genera *Hadromeros* Lane, 1971, *Parisoceraurus* Zhou in Zhou et al., 1977 and *Ceraurinium* Přibyl and Vaněk in Přibyl et al., 1985. *Ceraurinella magnilobata* is included within the “*Hadromeros* clade”, and consequently the genus *Ceraurinella* is resolved as polyphyletic. However, given that the aim of this work is not to unravel detailed relationships within genera, a more detailed study of *Ceraurinella* would be required to assess this possibility.

## Systematic palaeontology

**Family Cheiruridae Hawle and Corda,** 1847

**Subfamily Deiphoninae Raymond,** 1913

 = Cyrtometopinae Öpik, 1937

*Included genera*: *Actinopeltis* Hawle & Corda, 1847; *Cyrtometopus* Angelin, 1854 (= *Ancyginaspis* Přibyl and Vaněk in Přibyl et al., 1985); *Ellipsocoryphe* Lu, 1975; *Deiphon*, Barrande, 1850; *Hemisphaerocoryphe* Reed, 1896 (= *Cyrtometopella* Nikolaisen, 1961); ?*Hinggania* Zhao, Zhang, Cheng and Shu, 1997; *Junggarella* Xiang and Zhang in Zhang T., 1981; *Krattaspis* Öpik, 1937; *Mainbrookia* Adrain & Pérez-Peris, 2021; *Onycopyge* Woodward, 1880; *Reraspis* Öpik, 1937; *Sphaerocoryphe* Angelin, 1854; ?*Zazvorkaspis* Přibyl & Vaněk, 1964.

*Diagnosis*: Glabella moderately inflated with anterior margin anteriorly bowed; anterior border of cranidium dorsally overlapped by glabella and downturned from horizontal; S2 and S3 narrow (tr.) and transversally directed; thoracic pleural furrow slightly oblique at most proximal par, trasnverse distally; second pygidial axial ring not differentiated from pleural field by axial furrow.

*Discussion*: The results obtained suggest that “cyrtometopines” sensu Pärnaste (2003) are a paraphyletic grade at the base of a clade representing Deiphoninae. Consequently, “Cyrtometopinae” must be considered an invalid group and all the members previously assigned to it are included in Deiphoninae. These results contradict the hypothesis of Lane (1971, 2002), who included the “cyrtometopines” with an anteroposterior pleural constriction within Cheirurinae. Lane (1971, 2002) suggested that the thoracic pleural furrow of “cyrtometopines” and cheirurines are almost identical, though it is distinctively more transversely oriented in “cyrtometopines”. Lane (1971, 2002) also pointed out that the proximal part of the thoracic furrow in the genus *Cyrtometopus* runs obliquely from the anterior corner of the pleura as it does in cheirurines. However, in cheirurines the pleural furrow runs obliquely from the anterior corner of the pleura to the most distal region, where it then curves anteriorly. This configuration is completely different from the condition seen in deiphonines, which have a pleural furrow running transversely from the most proximal to the most distal part, or with only the most proximal part running obliquely as in *Cyrtometopus* and *Krattaspis*. Moreover, besides the pleural furrow there are other morphological characters that support the inclusion of “cyrtometopines” within Deiphoninae: the inflation of the glabella, the greatly bowed anteriorly anterior margin of the glabella, the downturned anterior border of the cranidium and the relatively short (sag.) hypostome. *Krattaspis popovi* Pärnaste, 2003 from the Mäeküla and Vassilkovo beds, Billingen Stage (Floian), St. Petesburg Region (Russia), is a perfect example of a “cyrtometopine” with strong similarities to more derived deiphonines. The glabella of *Krattaspis popovi* displays a transglabellar S1 (Pärnaste, 2003, Fig. 6. L-O, p. 248), which is a morphological characteristic of deiphonines such as *Sphaerocoryphe.* In addition, the anterior part of the glabella of *Krattaspis popovi* is more inflated than the rest of “cyrtometopines”, resembling the glabellar inflation found in *Hemisphaerocoryphe* or *Mainbrookia.* In summary, all of these morphological features combined with the phylogenetic results, favour the inclusion of “cyrtometopines” within Deiphoninae.

The idea of deiphonines as derived “cyrtometopines” has been suggested since Schmidt (1881), who in his pioneering early work proposed a link between *Cyrtometopus* and *Sphaerocoryphe* via the species *Hemisphaerocoryphe pseudohemicranium* (Nieszkowski, 1859). Subsequently, Öpik (1937) proposed Cyrtometopinae, recognizing the close link between the two subfamilies and the validity of both of the subfamilies as coherent groupings. This view, of deiphonines as derived “cyrtometopines” but recognizing the latter as a valid named group was followed by several subsequent authors (e.g., Pärnaste, 2003; Prantl & Přibyl, 1948; Přibyl et al., 1985). Pärnaste (2003, p. 245) pointed out the necessity of testing the validity of “Cyrtometopinae” within an analysis comparing them with the rest of cheirurid subfamilies. Finally, Adrain and Pérez-Peris (2021) suggested the paraphyly of “cyrtometopines” and their basal position to the deiphonines. The phylogenetic framework herein supports previous hypotheses of close relationship between Deiphoninae and “Cyrtometopinae”. However it rejects “cyrtometopines” as a monophyletic group, considering them as a paraphyletic grade.

**Tribe Deiphonini Raymond,** 1913

*Included genera*: *Deiphon*, Barrande, 1850; *Mainbrookia* Adrain & Pérez-Peris, 2021; *Onycopyge* Woodward, 1880; *Sphaerocoryphe* Angelin, 1854.

*Diagnosis*: One or two prefixigenal spines retained in holaspids; trunk with 13 segments; in late-diverging forms the last thoracic segment attached to the pygidium; pair of ventral projections on the pygidial rim.

*Discussion*: Deiphoninae encompasses a large range of morphological variability. The Ordovician deiphonines *Mainbrookina* and *Sphaerocoryphe* and the post-Ordovician taxa *Deiphon* and *Onycopyge* are morphologically highly distinct from the early diverging taxa within the subfamily. Consequently, the synapomorphies that define Deiphoninae have been transformed in the more derived forms. In order to characterise the deiphonine group that contains the post-Ordovician taxa and its most direct Ordovician relatives, the tribe Deiphonini is proposed. Both features are synapomorphies of the *Mainbrookia* + *Sphaerocoryphe* clade and they are found in younger deiphonines. In addition, the retention of the last thoracic segment in the holaspid pygidium is an apomorphy of *Sphaerocoryphe* also shared with post-Ordovician forms. These features experienced modifications in the shape in the post-Ordovician forms, as for example the prefixigenal spines are placed in a more ventral position, the anterior pygidial segment is no longer morphologically similar to a posterior thoracic segment and the posterior ventral projections are located in a more posterior and dorsal position than in *Mainbrookia* and *Sphaerocoryphe*. But here there can be no doubt that the structures present in post-Ordovician deiphonines are homologous with the structures present in *Mainbrookia* and *Sphaerecoryphe*. All these shared characters, together with the strong similarities in the protaspid morphology between *Sphaerocoryphe* and *Deiphon* (Chatterton, 1980; Chatterton & Perry, 1984), support the relationship of *Mainbrookia* and *Sphaerecoryphe* with the post-Ordovician forms and the inclusion of all of them in a formally named group.

## Discussion

This study provides the first quantitative phylogenetic framework for Cheirurinae and Deiphoninae, clarifying their deep nodes, identifying Ordovician clades within both subfamilies and mapping major morphological changes through the evolution of the group. A previous analysis of Deiphoninae performed by Congreve and Lieberman (2010) was focused on the relationships of the historically considered deiphonines, the genera *Hemisphaerocoryphe*, *Ellipsocoryphe* and *Sphaerocoryphe* and the Silurian genera *Deiphon* and *Onycopyge*, without considering early deiphonines. Comparison between results from both studies is not straightforward since the taxa included in each are considerably different. However, both analyses recovered *Hemisphaerocoryphe* and *Ellipsocoryphe* as sister taxa of *Sphaerocoryphe*.

**Fig. 6:**
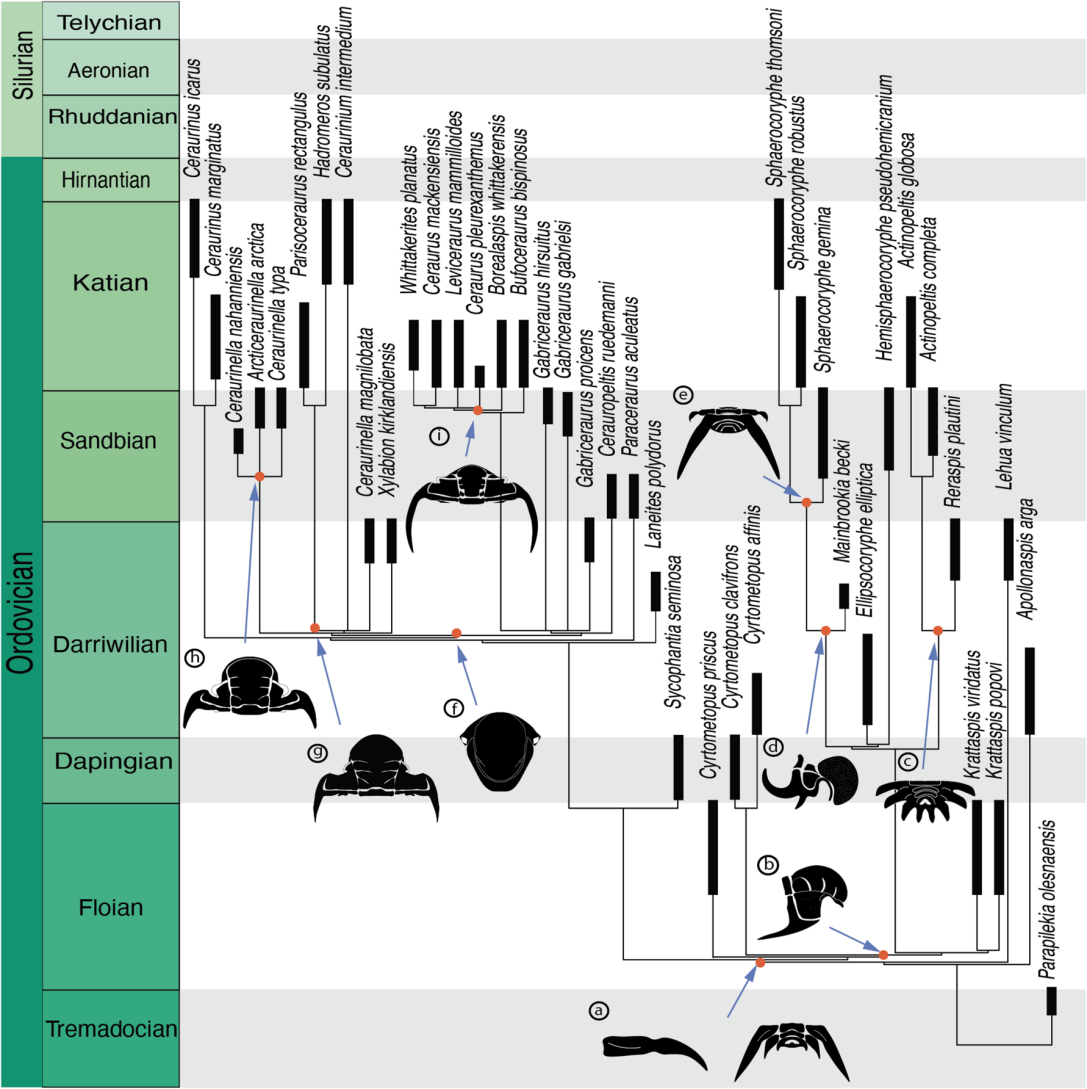
Time-scaled phylogeny from the strict consensus tree obtained from the parsimony analysis under equal weights. Black rectangles represent the approximate stratigraphical range of the species analysed. **a**–**i** schematic drawings representing morphological changes in the evolution of the group. **a** Thoracic pleura with anteroposterior constriction and pygidium with elongated anterior pair of pygidial spines. **b** Lateral view of cranidium showing moderate dorsal inflation of the glabella. **c** Dorsal view of pygidium with pygidial spines of similar length. **d** Lateral view of cranidium showing prefixigenal spines and effacement of S2 and S3. **e** Dorsal view of pygidium showing the retention of the last thoracic segment. **f** Ventral view of hypostome showing an anterior border greatly bowed forward and an elongated middle body. **g** Dorsal view of cranidium showing expanded frontal lobe and small palpebral lobes. **h** Dorsal view of a cranidium showing large palpebral lobes and relatively small genal spines. **i** Dorsal view of a cranidium showing small palpebral lobes and large genal spines.

The results obtained in the analysis are congruent with most of the previous hypotheses about the evolution of the group (e.g. Whittington, 1966). The position of *Apollonaspis arga* and *Lehua vinculum* are departures from previous opinion (e.g., Whittington, 1963; Pérez-Peris et al., 2021). Previous works have included both in Cheirurinae. The cranidial features of *Apollonaspis arga* (e.g., position and shape of the eyes, glabellar shape, glabellar furrows orientation) resemble those found in pilekiines. The thorax and hypostome of *A. arga* are unknown. Whittington (1963, pl. 23, figs. 5, 7) questionably assigned a pygidium to the species, but it almost certainly belongs to one of the cooccurring heliomerine species (to which Whittington [1963, pl. 25, fig. 3] misassigned an acanthoparyphine pygidium). Hence, the lack of data for any sclerite beyond the cranidium and librigenae, together with the strong resemblance of its cephalon with the cephalon of pilekiines explain the position of the species outside the Cheirurinae + Deiphoninae clade. Material of the thorax and pygidium is needed in order to reconsider the position of *Apollonaspis arga* with confidence. In the case of *Lehua vinculum,* all sclerites except the hypostome and rostral plate are known. The thoracic pleural furrow of *L. vinculum* is situated in the proximal part of the pleura (from the axis to the fulcrum) running obliquely from the proximal anterior corner, as observed in the species belonging to Cheirurinae (Pérez-Peris et al., 2021). In addition, the trunk is divided into a thorax with 11 segments and a pygidium with 3 segments as in the rest of cheirurines. Both of these characteristics suggest a strong affinity with cheirurines. However, the new phylogenetic hypothesis suggests that the morphology of the thoracic pleural furrow and trunk segmentation typical from cheirurines are symplesiomorphic to the Cheirurinae + Deiphoninae clade. The thoracic pleurae of *L. vinculum* are not constrained at the fulcrum and the pygidial spines are all of similar size. The anteroposterior constriction of the pleura and the pygidium configuration with a macropleural spine are synapomorphies of Cheirurinae + Deiphoninae (Fig. 6). Therefore, *Lehua vinculum* appears to lie outside this clade.

When the stratigraphic ranges of the different species are considered, general patterns about the timing of evolution of the group can be explored (Fig. 6). The oldest members assigned to either subfamily date from the Floian. Within Cheirurinae, *Sycophantia* sp*.* (Adrain & Fortey, 1997) from the Tourmakeady Formation, County Mayo, Ireland, is the oldest putative cheirurine. However, the material of *Sycophantia* sp*.* is very fragmentary, and it was impossible to include the species in the analyses. *Sycophantia seminosa* from the Valhallfonna Formation, Spitsbergen, Svalbard (Dapingian), is the oldest species with well-preserved morphological information. Within Deiphoninae, the Floian *Cyrtometopus priscus* from the Latorp Limestone, Närke, Sweden and *Krattaspis viridiatus* Öpik, 1937 from the Mäeküla Formation, Estonia, are the oldest members assigned to the subfamily. Consequently, the split between these subfamilies happened during the Early Ordovician and by the Floian both clades were established (Fig. 6). In both subfamilies, fossils from the major lineages within each clade are already present by the Darriwilian. Within Cheirurinae, the Scottish *Ceraurinella magnilobata* and *Gabriceraurus proicens,* both from the Stinchar Limestone Formation, and *Xylabion kirkdandiensis* from the Superstes Mudstone Formation date from the Darriwilian. Within Deiphoninae, *Ellipsocoryphe elliptica* Lu, 1975 from the Meitan Formation, Sichuan, China, and *Mainbrookia becki* Adrain & Pérez-Peris, 2021 from the Table Cove Formation, Newfoundland, Canada, also date from the Darriwilian. These temporal ranges suggest that during the Floian–Darriwilian period, the major lineages within Cheirurinae and Deiphoninae were established (Fig. 6).

### Major morphological patterns in the evolution of Deiphoninae and Cheirurinae

Deiphoninae experienced major morphological changes throughout its Ordovician evolutionary history, with derived forms, such as the genus *Sphaerecoryphe*, showing several features markedly different from earlier taxa. Morphological differentiation between basal and derived forms was one of the causes that led to the classification of “cyrtometopines” as a group separate from deiphonines. However, once all the morphological characters of the group are taken in to account, it is possible to trace the main morphological changes through the history of the clade.

During the evolution of the group, changes in the glabella are particularly pronounced, including dramatic inflation. Early forms display a flat (“*Cyrtometopus*” *priscus*) or moderately inflated (*Cyrtometopus* and *Kratttaspis*) glabella (Fig. 6b), while in later forms it is highly inflated (e.g. *Actinopeltis*, *Mainbrookia*, *Sphaerocoryphe*) (Fig. 6d). The ‘bubble-headed’ morphotype has evolved multiple times independently within Trilobita, and three different morphotypes have been identified (Fortey & Owens, 1997). One morphotype corresponds to the inflation of the preglabellar field (e.g., *Parabolinella* Brögger, 1882), another with inflation of the anterior lobe of the glabella (e.g., *Staurocephalus* Barrande, 1846) and the last to the inflation of the whole glabella except the basal lobes (L1) (e.g., *Deiphon*). Inflation in *Sphaerocoryphe* represents the last morphotype and it has been proposed that the inflated glabella reflects an increase in stomach capacity (Fortey & Owens, 1997). Other modifications associated with the glabella are the effaced lateral glabellar furrows S2 and S3 (Fig. 6d), the reduction of basal glabellar lobe L1 (Fig. 6d), and a stronger arching of the anterior border of the cranidium.

In Deiphoninae, paedomorphosis appears to have played an important role in the evolution of the group, with several juvenile characters retained in adults of *Sphaerocoryphe* (Adrain & Pérez-Peris, 2021; p. 11). The paedomorphically retained characters in *Sphaerocoryphe* include: (1) The prefixigenal spines. The prefixigenal spines are well developed in the cheirurid protaspid (e.g., Whittington & Evitt 1954, Chatterton, 1980). In most cheirurids the first and the second anterior pair of spines are reduced during development, disappearing completely in the adult, while the most posterior spine forms the genal spine. Acanthoparyphines are an exception, with some members of the subfamily retaining the prefixigenal spines in adults. In *Sphaerocoryphe* and *Mainbrookia* the prefixigenal spines, one or two pairs, are retained (Fig. 6d). (2) The anteriorly placed eyes. In the trilobite protaspid the eyes are located in an anterior position and during development tend to move to a more posterior position in the cheek (e.g., Chatterton, 1980; Whittington & Evitt, 1954). In *Sphaerocoryphe* adults, the eyes are relatively anteriorly positioned. (3) The reduced number of trunk segments, and the retention of a thoracic segment in the pygidium. The symplesiomorphic configuration of Deiphoninae is 14 segments in the thoracopygidium, of which 11 are allocated to the thorax and 3 to the pygidium. Such a condition is present in *Cyrtometopus* and *Krattaspis*. In *Hemisphaerocoryphe* the total number of segments in the thoracopygidium is reduced to 13, with 10 in the thorax and 3 in the pygidium. The same number thoracopygidial segments (13) are found in *Sphaerocoryphe* but in the adult the last thoracic segment is retained in the pygidium, resulting in a configuration of 9 thoracic segments and 4 pygidial segments (9 + 4) (Fig. 6e). During development trilobites form new segments in the transitory pygidium (anamorphic phase) and release them into the thorax (Hughes, 2003). A reduction of the number of segments in the trunk implies a shorter anamorphic phase, while the retention of a segment in the pygidium indicates the retention of the last meraspid stage into the adult form.

Paedomorphosis has been widely described across trilobites and identified as a major driver for morphological innovations, especially within Cambrian forms (see McNamara[1986] for a summary). Several examples have been described of reduction of the number segments of the trunk and thoracic segments (McNamara et al., 2003, 2006). In post-Ordovician trilobites fewer examples of paedomorphosis have been described (e.g., *Encrinurus* Emmrich, 1844; Edgecombe & Chatterton, 1987). Peramorphosis has been proposed as predominant in the evolution of the group (McNamara, 1986). Deiphoninae is a good example of paedomorphic evolution in a lineage of post-Ordovician trilobites. However, to better understand the role of heterochronic processes in the evolution of the group, it would be necessary to find more complete ontogenetic series of basal deiphonines.

Throughout the evolution of Cheirurinae the body plan of the group remained relatively similar, as compared with the significant changes within Deiphoninae. Within Cheirurinae the number of segments in the thoracopygidium (11 + 3) remains stable across the whole group. The shape of the glabella, the development of the pygidial spines and the surface sculpture are the morphological features that experience the most changes. The ‘*Ceraurus*-like’ lineage is characterised by the development of a long and stout genal spine (Fig. 6i) and first pygidial pleural spine. Late-diverging forms experienced a reduction of the last two pairs of pygidial spines. In taxa such as *Whittakerites planatus* and “*Ceraurus” mackenziensis*, the posterior part of the pygidium (posterior to the anterior elongate pygidial spine) is dramatically reduced with the marginal spines lost. Furthermore, the ‘*Ceraurus*-like’ lineage is characterised by significant changes in sculpture, especially of the glabella. The genera *Cerauropeltis* and *Gabriceraurus* have granular sculpture. *Bufoceraurus* Hessin, 1989 and *Borealaspis* Ludvigsen, 1976 develop coarser tubercles on the surface of the glabella, while later-diverging taxa within the clade reduced or lost the glabellar ornamentation (e.g., *Whittakerites* Ludvigsen, 1976). Through the evolution of the ‘*Ceraurinella*-like’ clade, changes occur in the shape of the glabella and the size of the pygidial pleural spines. The clade containing *Hadromeros* is characterised by the expansion of the anterior lobe of the glabella and the increase in width (tr.) of S2 and S3 (Fig. 6g). The size of the pygidial spines is variable across the whole group, from forms where all pygidial spines are sub-equal in length, such as in *Hadromeros*, to forms where the first pair is much longer than the rest, such as *Parisoceraurus*.

Members of the ‘*Ceraurinella*-like’ clade were previously suggested as ancestors for Silurian and Devonian cheirurines (e.g., Chatterton & Perry, 1984; Lane, 1971). Such hypothesis is based on the morphological similarities between the Ordovician *Ceraurinella*, *Hadromeros* and *Xylabion* and the post-Ordovician cheirurines, particularly the Silurian forms*.* Silurian genera such as *Ktenoura* Lane, 1971, *Proromma* Lane, 1971, *Contracheirurus* Chatterton & Perry, 1984 and *Didrepanon* Lane, 1971 are characterised by: a slightly expanded anterior glabellar lobes; S1 running obliquely posteromedially proximally; smooth (or finely granulated) glabellar surfaces; relatively short genal spines; hypostomes with well-defined maculae and transverse posterior border; pygidia with all the genal spines sub-equally developed and in some species the anterior pygidial flange enlarged. All these morphological characteristics are shared with species from the ‘*Ceraurinella*-like’ clade and absent in those from the ‘*Ceraurus*-like’ clade. Moreover, *Hadromeros* is the only presently recognised cheirurine genus that crosses the Ordovician–Silurian boundary (Lane, 1971). Lane (1971, p. 74, Fig. 10), proposed *Xylabion* as ancestor to *Hadromeros,* although he expressed uncertainty. Chatterton and Perry (1984) suggested *Ceraurinella* as a possible *Hadromeros* ancestor, owing to the similar pygidial morphology between both genera. In the present analysis, *Ceraurinella magnilobata* is resolved as sister taxon to the clade containing *Hadromeros*. Despite the different taxa suggested as a *Hadromeros* ancestor, all hypotheses agree that the post-Ordovician cheirurines are related to the ‘*Ceraurinella*-like’ lineage, which extended into the Silurian and subsequently diversified. Even though further analyses including Silurian and Devonian forms are needed, the position of the ‘*Ceraurinella*-like’ clade as a basal grade to the Silurian and Devonian cheirurines is here supported.

## Conclusions

1. Multiple congruent phylogenetic analyses resolved the subfamily “Cyrtometopinae” as a paraphyletic grade at the base of a major clade that includes the members of the subfamily Deiphoninae. Consequently, “Cyrtometopinae” is considered a junior subjective synonym of Deiphoninae.
2. Cheirurinae and Deiphoninae form a major clade within the family Cheiruridae, primarily defined by the anteroposterior constriction of the thoracic pleura. The split between both clades occurred somewhere in the Early Ordovician. By the Darriwilian all major lineages of both subfamilies were already established.
3. Paedomorphosis plays an important role in the evolution of Deiphoninae. The genus *Sphaerocoryphe* retains several juvenile characters in the adult forms. Such characters are fixed in the post-Ordovician forms *Deiphon* and *Onycopyge*.
4. Within Cheirurinae two major clades can be identified, consisting of the ‘*Ceraurus*-like’ and ‘*Ceraurinella-*like’ clades. The post-Ordovician forms share more morphological similarities with members of the ‘*Ceraurinella*-like’ clade, suggesting that younger cheirurines could be descendent from this lineage.

## Supplementary Information

Supplementary Material 1.

Supplementary Material 2.

Supplementary Material 3.

Supplementary Material 4.

Supplementary Material 5.

Supplementary Material 6.

## Data Availability

Data is provided within the manuscript or supplementary information files.
